# Sex differences in social buffering and social contagion of alarm responses in zebrafish

**DOI:** 10.1007/s10071-023-01779-w

**Published:** 2023-05-15

**Authors:** Ibukun D. Akinrinade, Susana A. M. Varela, Rui F. Oliveira

**Affiliations:** 1 IGC–Instituto Gulbenkian de Ciência, Rua Quinta Grande 6, Oeiras, Portugal; 2 Present Address: HBI-Hotchkiss Brain Institute, Cummings School of Medicine, University of Calgary, Alberta, Canada; 3 WJCR–William James Center for Research, ISPA–Instituto Universitário, Lisbon, Portugal; 4 ISPA–Instituto Universitário, Lisbon, Portugal; 5 Champalimaud Neuroscience Programme, Champalimaud Centre for the Unknown, Lisbon, Portugal

**Keywords:** Alarm substance, Life-history strategies, Social buffering, Social contagion, Sex differences, Zebrafish

## Abstract

The alarm substance in fish is a pheromone released by injured individuals after a predator attack. When detected by other fish, it triggers fear/defensive responses, such as freezing and erratic movement behaviours. Such responses can also help other fish in the shoal to modulate their own behaviours: decreasing a fear response if conspecifics have not detected the alarm substance (social buffering) or triggering a fear response if conspecifics detected the alarm substance (social contagion). Response variation to these social phenomena is likely to depend on sex. Because males have higher-risk life-history strategies than females, they may respond more to social buffering where they risk not responding to a real predator attack, while females should respond more to social contagion because they only risk responding to a false alarm. Using zebrafish, we explored how the response of males and females to the presence/absence of the alarm substance is modified by the alarmed/unalarmed behaviour of an adjacent shoal of conspecifics. We found that, in social buffering, males decreased freezing more than females as expected, but in social contagion males also responded more than females by freezing at a higher intensity. Males were, therefore, more sensitive to visual information, while females responded more to the alarm substance itself. Because visual information updates faster than chemical information, males took more risks but potentially more benefits as well, because a quicker adjustment of a fear response allows to save energy to other activities. These sex differences provide insight into the modifying effect of life-history strategies on the use of social information.

**Supplementary Information:**

The online version contains supplementary material available at 10.1007/s10071-023-01779-w.

## Introduction

Adaptive behaviour relies upon the appropriate response to information from a variety of sources, particularly when the information concerns threats to survival (e.g., the presence of a predator). Timely risk-assessment and adequate risk-avoidance behaviours are pivotal (Lima and Dill 1990), although individuals may vary in their propensity to be at risk (Réale et al. 2007). Sex-specific differences in risk-taking behaviour are well documented (Schuett et al. 2010), with males generally reported to be bolder and, hence, higher risk-takers than females (Harris et al. 2010; King et al. 2013; Roy et al. 2017). One reason for this may be that, in many species, high-risk taking behaviour provides males with higher rewards that may confer competitive advantages over food, territories or mates, while females, because they generally invest more in reproduction, favour safety (Jolles et al. 2015; King et al. 2013; Spence et al. 2008; Trivers and Campbell 2010).

In social species, apart from using direct cues from danger sources (e.g., sound produced by a predator before engaging in an attack), individuals can also use information from conspecifics, such as alarm signals (e.g., alarm calls: (Gill and Bierema 2013)) or defensive behaviours (e.g., fleeing: (Morelli et al. 2019); and freezing: (Pereira et al. 2012)), to assess the presence of danger. This is the case of the alarm substance and the defensive/fear responses that it elicits in fish. The alarm substance (aka *Schreckstoff*) is a chemical cue, a pheromone, that is released from epidermal club cells upon injury of the skin and that is known to be produced by many species of *Ostariophysi* fish (Smith 1992), a super-order that includes the zebrafish *Danio rerio*. It prompts an alarm or fear response that has been interpreted as adaptive in the context of a predator attack (Chivers and Smith 1998; Pfeiffer 1977; Frisch 1942). In zebrafish, the characteristic defensive/fear response includes erratic movement (making multiple darts and fast acceleration bouts in rapid succession in which the direction of movement also changes in a stochastic manner) and freezing (complete cessation of movement at the bottom of the water column, except for the movement of the operculum and eyes) (Gerlai et al. 2000, 2006; Kalueff et al. 2013; Speedie and Gerlai 2008; Waldman 1937). Erratic movement has been suggested to act as a predator deterrent by visually confusing predators (also known as “protean behavior”, Humphries and Driver 1970) due to the high speed and stochastic swimming associated with these motor patterns (Parra et al. 2009); while freezing has been reported to be a form of attentive immobility serving to avoid detection by predators and to enhance perception (Roelofs 2017).

The alarm substance and the defensive/fear responses it elicits provide valuable information to other fish in the shoal about the level of danger they are facing (Debiec and Olsson 2017; Kikusui et al. 2006; Oliveira and Faustino 2017; Speedie and Gerlai 2008). However, the alarm substance can sometimes be ambiguous because it can remain invariable in the water for long periods of time, including after the predator is gone, or it may not reach all group members (Oliveira and Faustino 2017; Stephenson 2016). On the other hand, alarm responses from conspecifics may occur by mistake (for example, when the individual bruises its skin against a rocky surface), and vary among individuals (Pinho et al. 2020). This means that the two sources of social information are not completely reliable and may often conflict with each other: fish that have joined a shoal may detect the alarm substance but observe non alarmed conspecifics, or do not detect the alarm substance but observe alarmed conspecifics. Certainty about the level of risk is reduced in these situations and the difficulty is to decide whether to respond or not to a possible threat. We ask if this decision differs between males and females.

When the information provided by unalarmed conspecifics conflicts with the direct detection of the alarm substance by the individual, there is uncertainty about the *presence* of a threat; deciding that the threat is absent in such situations leads to dropping a defence strategy (social buffering of fear), although not without the risk of missing a true attack (Oliveira and Faustino 2017). We predict that males, because they are higher risk takers, are more likely to respond to social buffering than females. Likewise, when the information provided by alarmed conspecifics conflicts with the non-detection of the alarm substance by the individual, there is uncertainty about the *absence* of a threat; deciding that the threat is present in such situations leads to adopting a defensive behaviour (social contagion of fear), even though the individual may be responding to a false alarm (Oliveira and Faustino 2017). We predict that females, because they are lower risk takers, are more likely to respond to social contagion than males.

The social phenomena of buffering and contagion of fear are highly conserved and possibly share similar neural mechanisms (Kikusui et al. 2006; Oliveira and Faustino 2017). They have been reported in birds (Edgar et al. 2015), mice (Panksepp and Lahvis 2016; Gutzeit et al. 2019), rats (Davitz and Mason 1955; Kim et al. 2010; Jones et al. 2014; Kiyokawa et al. 2014, 2018; Fuzzo et al. 2015), sheep (Da Costa et al. 2004; González et al. 2013), monkeys (Kalin and Shelton 1989; Mineka et al. 1984), and humans (Haaker et al. 2017; Gunnar 2017). This implies that using fear responses from conspecifics to modulate defence strategies must be adaptive to both males and females. What is unknown is whether males and females differentially use conspecifics’ fear responses to modulate their own response thresholds.

Social buffering of fear has previously been reported to occur in zebrafish males (Faustino et al. 2017; Mathuru et al. 2017); and to occur independently of shoal size (two, four or eight) (Faustino et al. 2017). Groups of zebrafish that could see a group of alarmed demonstrators were also reported to show social contagion with intense and prolonged alarm reactions (Hall and Suboski 1995; Suboski et al. 1990), as well as individual fish were reported to show social contagion from a single alarmed demonstrator, especially if the demonstrator was a familiar conspecific (Fernandes Silva et al. 2019). However, the assessment of possible sex differences in zebrafish or other species responses to social buffering and social contagion of fear has not been tested yet. In the present work, we have used an experimental behavioural approach that allowed the investigation of sex differences in social buffering and social contagion of fear in zebrafish.

## Methods

### Animals and housing

All zebrafish (*Danio rerio*) used in this study were 6–12 months old and came from the Tuebingen (TU) wild-type strain. They were bred and raised at the fish facility of Instituto Gulbenkian de Ciência (IGC, Oeiras, Portugal) in mixed-sex 5 L tanks (n = 35 per tank) in a recirculating system (ZebTEC active-blue, Tecniplast) until they were about 2 months old, then transferred to 120-capacity stock tanks (25 L) until ready for experiments. Fish were kept under 14L: 10D photoperiod, tank water was maintained at 28 ºC, 900 μS, pH 7.0, < 0.2 ppm nitrites, < 50 ppm nitrates and 0.01–0.1 ppm ammonia. Fish were fed twice a day with *Artemia salina* and commercial food flakes (Bionautic). Both test fish, stimuli fish and alarm substance donors were bred from same group of breeders.

### Alarm substance extraction

Alarm substance (AS) was extracted using a modified version of the protocol described by Speedie and Gerlai (2008). Ten donor zebrafish (five males and five females) were used to prepare the alarm substance for the experiment as a one-time batch, after which aliquots of 1 ml were stored. Donor fish were retrieved from the holding tank with a fishing net, and then quickly sacrificed, placed in a Petri dish on ice and 15 shallow cuts were made on both sides of the fish using a sterile surgical blade. Adequate release of AS was ensured by washing the cuts with 50 ml of distilled water (vehicle) using a Terumo® syringe (8SS50L1) without a needle. Impurities were removed from the AS solution using 0.22 μm sterile vacuum filtering (Filtropur V50- 83.3940) and AS aliquots were stored at − 20 °C.

### Behavioural setup

The behavioural setup established by Faustino et al. (Faustino et al. 2017) was adopted, with two adjacent tanks (12 × 12 × 15 cm), each filled with 1.3 L of water. We placed individual test fish in one tank, and the other tank contained water with or without a shoal of conspecifics (two males and two females). The tanks had white opaque bases, opposite and rear walls to prevent interference by external environmental cues. AS or vehicle (distilled water) were administered with a flexible and transparent PVC tubing (0.8 mm internal diameter; 2.4 mm external diameter). Two video cameras (either a pair of B&W mini surveillance cameras (Henelec 300B) or two webcams (Logitech C 525 high-definition camcorder, Ref 960-000842)) with an acquisition rate of 30 fps were placed above and to the side of the tanks. Video acquisition was done with Pinnacle Studio 14 (Corel Corporation, Ottawa, Canada) software and EyeLine Video Surveillance software (www.nchsoftware.com).

### Experimental procedure

**Fig. 1:**
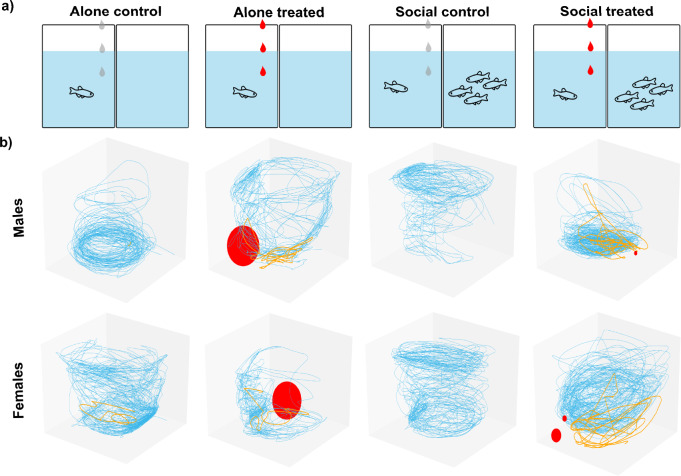
Social buffering paradigm. **a** Schematic representation of the behavioural treatments. Droplets represent administration of vehicle (grey) and alarm substance (AS; red) to control and experimental groups. **b** Representative 3D swimming behaviour for each treatment group in males and females: normal swimming pattern (blue), erratic movement (orange), and freezing episodes (red dots, where size is proportional to freezing time) (colour figure online)

Social buffering and social contagion of fear were tested in two separate experiments. In social buffering, test fish were randomly assigned to one of four treatments based on the presence/absence of the AS and on the presence/absence of a shoal: test fish alone exposed to vehicle (Alone Control); test fish alone exposed to AS (Alone Treated); test fish exposed to vehicle in the presence of a shoal (Social Control); and test fish exposed to AS in the presence of a shoal (Social Treated) (Fig. 1).

**Fig. 2:**
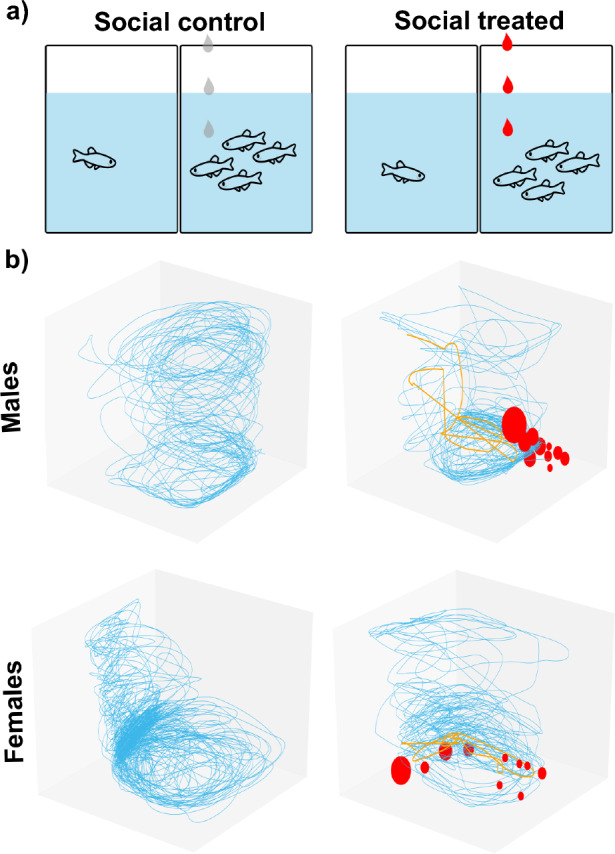
Social contagion paradigm. **a** Schematic representation of the behavioural treatments. Droplets represent administration of vehicle (grey) and alarm substance (AS; red) to control and experimental groups. **b** Representative 3D swimming behaviour for each treatment group in males and females: normal swimming pattern (blue), erratic movement (orange), and freezing episodes (red dots, where size is proportional to freezing time) (colour figure online)

In social contagion, test fish were randomly assigned to one of two treatments based on the presence/absence of the AS and always in the presence of a shoal: test fish with shoal exposed to vehicle (Social Control); and test fish with shoal exposed to AS (Social Treated) (Fig. 2). The order of testing was randomized in each experiment to control for possible effects of time of day and all experiments were conducted between 10:00 and 15:00. In social contagion, we made sure the stimuli shoal exposed to the AS displayed a clear alarm response that consisted in erratic movement bouts alternating with freezing.

Shoals used as a social stimulus in both experiments were used only once. They were made up of two males and two females, which were familiar with each other (i.e., always retrieved from the same stock tank), but test fish were unfamiliar with the shoal fish (i.e., test fish were always taken from a different stock tank to that of the stimuli fish). Test animals were also tested only once after which they were euthanized. In social buffering, we used 80 males (20 animals per experimental condition) and 80 females (20 animals per experimental condition) as test fish, and in social contagion, we used 44 males (24 for social control and 20 for social treated experimental conditions), and 45 females (25 for social control and 20 for social treated experimental conditions). In the two experiments together, we used 169 independent shoals (84 males and 85 females).

On the day before the experiment, male and female test fish were taken from home tanks and placed in testing apparatus overnight, for acclimation. For alone treatment groups, test fish were kept in a tank alone and an empty tank was placed adjacent to it. For the social treatment groups, test fish were placed in a tank and stimuli fish were placed in the adjacent tank overnight for acclimation to both the shoal and tanks. On the test day, each trial lasted for 15 min and started with a 5-min baseline recording (Baseline phase). The experimental fish in social treatment groups could see the stimuli fish during the baseline recording phase. Then, the AS or vehicle was administered following the protocol of Speedie and Gerlai (2008), and test fish behaviour was recorded for 10 min (Test phase). In brief, 0.8 mL of distilled water (vehicle) and 0.8 mL of AS were delivered using a 5 mL Terumo® syringe. The syringe was connected to a flexible and transparent PVC tubing. The tubing was hooked to the top of the tank and was allowed to go in the water to prevent ripples from disturbing the fish during AS or vehicle administration. The tanks had white opaque bases, opposite and rear walls to prevent interference by the experimenter. After the experiments, the tanks were cleaned using filtered water several times, after which they were left to dry and used to prepare the next day of experiments. Eight tanks were tested per day and two tanks were tested simultaneously, leaving a relatively short time interval between tests.

Given that the alarm substance degrades quickly (approximately 30 min; Chivers et al. 2013), it was kept in ice between trials and, therefore, the control (vehicle) was kept in the same conditions to ensure comparability of treatments. The arena dimensions for top and side views were 10.5 × 10.5 cm and 11 × 10.5 cm respectively. Two videos corresponding to the top and side view recordings were analysed per test fish. The x,y,z coordinates extracted from each frame using an automated video tracking software (Ethovision XT 12, Noldus Inc. Netherlands) were analysed using an updated version of custom made xyz2b Python scripts (https://github.com/joseaccruz/xyz2b). Percentage of time spent in erratic movement and freezing were used as measures of the fear response in the experiments. The erratic movement was defined by two criteria; if the test fish accelerated > 8 cm/s^2^ and > 5 changes in direction/sec. Freezing was defined by two criteria: if the test fish velocity was < 0.2 cm/s; and if the test fish position on y-axis was below the bottom quarter of the side view of the arena.

### Statistical analysis

Logarithmic transformations did not normalize the data from the three experiments, so the response variables (the percentage of time spent freezing and in erratic movement) were analysed using Generalized Linear Models (GLM) with beta regression (Cribari-Neto and Zeileis 2010). Because both the percentage of time spent freezing and in erratic movement vary continuously between 0 and 1, as required for beta regression, but assume sometimes the extreme value of 0, we transformed the data using the formula (y (n − 1) + 0.5)/n where n is the sample size (Smithson and Verkuilen 2006). The models included three explanatory variables and their double and triple interactions: Experimental Phase (with two groups, Baseline and Test phase), Sex (with two groups, Male and Female) and Treatment. For social buffering, Treatment had four groups (Alone Control, Alone Treated, Social Control, and Social Treated; Fig. 1) and for social contagion it had two groups (Social Control and Social Treated; Fig. 2).

Statistical analyses were performed using the R-programming software, version 4.0.2 (R Core Team 2020) with the following packages: betareg (for GLM with beta regression (Cribari-Neto and Zeileis 2010)) and emmeans (for extracting full effects within the betareg using the *joint_tests* function and for planned comparisons using the *emmeans* function with the false discovery rate (FDR) adjustment method (Russell et al. 2020)). p < 0.05 was assumed as the criterion for statistical significance throughout. The software Graphpad Prism version 8 was used to make the graphics. The software Inkscape version 0.92.3 was used to make the other figures.

## Results

### Sex differences in social buffering

The freezing response of test fish was significantly affected by the Experimental phase (F = 128.632, p < 0.0001), the Treatment (F = 61.927, p < 0.0001), and by the Sex of the test fish (F = 4.083, p = 0.0433). In addition, there was a significant interaction of Experimental phase and Treatment (F = 62.288, p = 0.0043), Experimental phase and Sex (F = 8.170, p = 0.0043), and Treatment and Sex (F = 5.382, p = 0.0011). The triple interaction was not significant (F = 2.254, p = 0.0799).

**Fig. 3:**
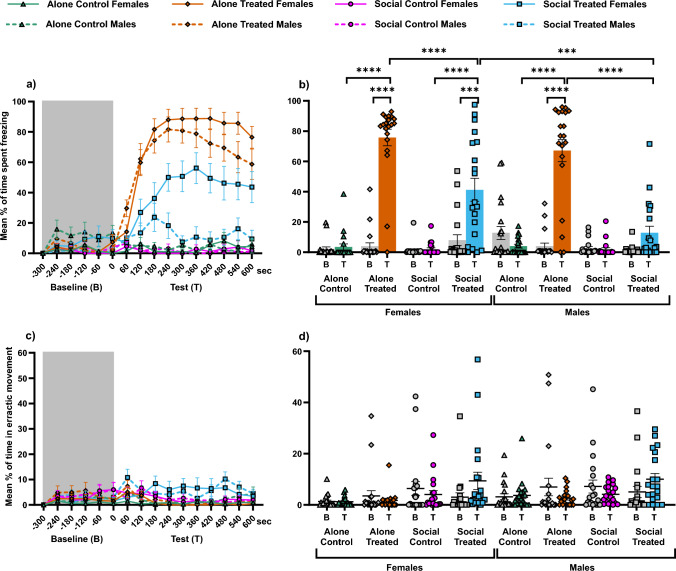
Zebrafish response in social buffering. **a** Temporal dynamics of freezing response across treatments. **b** Percentage of freezing (mean + SEM) before and after either vehicle or AS administration. **c** Temporal dynamics of erratic movement response across treatments. **d** Percentage of erratic movement response (mean + SEM) before and after either vehicle or AS administration. Shaded areas in **a** and **c** indicate time before AS or vehicle administration (Baseline, B). Non-shaded areas indicate time after AS or vehicle administration (Test phase, T). Gray bars and data points in **b **and **d** indicate, again, the Baseline (B) and coloured bars and data points indicate the Test phase (T) for each treatment: green and triangles for “Alone Control”, orange and diamond for “Alone Treated”, pink and circles for “Social Control”, and blue and squares for “Social Treated. In **a** and **c**, females are represented by solid lines and males by dashed lines. Asterisks indicate statistical significance: ***p < 0.001 and ****p < 0.0001 (colour figure online)

Although male and female test fish showed similar freezing responses to AS when alone (planned comparisons: Male Alone Treated vs Female Alone Treated: z = 1.094, p = 0.6085), males spent significantly less time freezing than females when exposed to AS in the presence of a shoal (planned comparisons: Male Social Treated vs Female Social Treated: z = 3.765, p = 0.0006). Nonetheless, both males and females reduced the freezing response when exposed to AS in presence of a shoal compared to when they were alone (planned comparisons: Male Social Treated vs Male Alone Treated: z = 7.884, p < 0.0001; Female Social Treated vs Female Alone Treated: z = 4.791, p < 0.0001). Additionally, AS significantly increased the freezing response of test fish when alone compared with control, independent of the sex (planned comparisons: Male Alone Treated vs Male Alone Control: z = − 8.791, p < 0.0001; Female Alone Treated vs Female Alone Control: z = − 11.423, p < 0.0001) and, as expected, control groups showed no significant difference in freezing response, independent of sex (planned comparisons: Male Social Control vs Male Alone Control: z = 0.698, p = 0.7717; Female Social Control vs Female Alone Control: z = − 0.021, p = 0.9831). Moreover, both male and female freezing responses to AS when alone were significantly higher than during the Baseline phase (planned comparisons: Male Alone Treated Baseline vs Male Alone Treated Test: z = − 8.705, p < 0.0001; Female Alone Treated Baseline vs Female Alone Treated Test: z = − 11.098, p < 0.0001). Female freezing responses to AS when with a shoal was also significantly higher than during the Baseline phase (planned comparisons: Female Social Treated Baseline vs Female Social Treated Test: z = − 4.275, p = 0.0001), but the males’ response was not (planned comparisons: Male Social Treated Baseline vs Male Social Treated Test: z = − 1.286, p = 0.4965). See Fig. 3a and b.

The erratic movement response of test fish was significantly affected by the Experimental phase (F = 5.068, p = 0.0244) and by Treatment (F = 3.208, p = 0.0221), but not by Sex (F = 3.606, p = 0.0576). The interactions between Experimental phase and Treatment (F = 2.282, p = 0.0769), Experimental phase and Sex (F = 0.099, p = 0.7526), and Treatment and Sex (F = 0.129, p = 0.9427) were not significant. The triple interaction was not significant either (F = 0.010, p = 0.9987).

Independently of Sex, there was no increase in erratic movement response to AS when alone (planned comparisons: Male Alone Treated vs Female Alone Treated: z = − 0.498, p = 0.9471), as well as when in the presence of a shoal (planned comparisons: Male Social Treated vs Female Social Treated: z = − 0.454, p = 0.9471). Both male and female test fish did not spend significantly more time in erratic movement when exposed to AS in the presence of a shoal than when exposed alone to AS (planned comparisons: Male Social Treated vs Male Alone Treated: z = − 2.171, p = 0.2491; Female Social Treated vs Female Alone Treated: z = − 2.197, p = 0.2491). Independently of sex, there was no increase in erratic movement response of the test fish when exposed to AS alone compared to the control treatment (planned comparisons: Male Alone Treated vs Male Alone Control: z = 0.444, p = 0.9471; Female Alone Treated vs Female Alone Control: z = − 0.190, p = 0.9843) and, as expected, control groups showed no significant difference in erratic movement independent of sex (planned comparisons: Male Social Control vs Male Alone Control: z = − 0.705, p = 0.9337; Female Social Control vs Female Alone Control: z = 0.867, p = 0.9337). Moreover, both male and female erratic movement responses were similar across treatments between Baseline and the Test phase (all p values > 0.05). See Fig. 3c and d.

Together, our observations demonstrate that when males and females were exposed to the alarm substance in the presence of a non-alarmed shoal, they decreased the proportion of time spent freezing relative to when exposed while alone, but males significantly more than females and there were no significant changes in erratic response in males and females.

### Sex differences in social contagion

There was a significant effect of Experimental phase (F = 88.098, p =  < 0.0001), Treatment (F = 111.805, p =  < 0.0001) and Sex (F = 5.391, p = 0.0202) on the freezing response of test fish and a significant interaction of Experimental phase and Treatment (F = 95.659, p < 0.0001), Experimental phase and Sex (F = 5.045, p = 0.0247), Treatment and Sex (F = 9.252, p = 0.0024), as well as their triple interaction (F = 5.588, p = 0.0181).

**Fig. 4:**
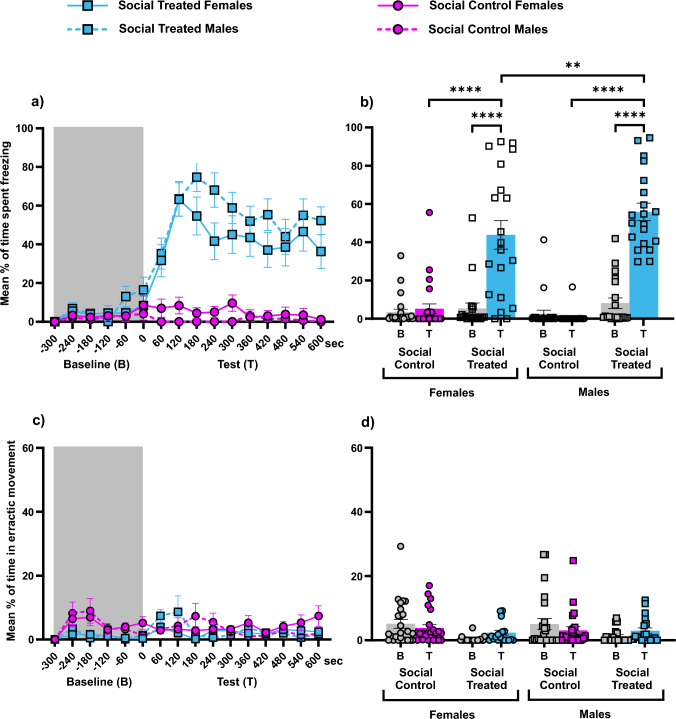
Zebrafish response in social contagion. **a** Temporal dynamics of freezing response across treatments. **b** Percentage of freezing (mean + SEM) before and after either vehicle or AS administration. **c** Temporal dynamics of erratic movement response across treatments. **d** Percentage of erratic movement response (mean + SEM) before and after either vehicle or AS administration. Shaded areas in **a** and **c** indicate time before AS or vehicle administration (Baseline, B). Non-shaded areas indicate time after AS or vehicle administration (Test phase, T). Gray bars and data points in **b** and **d** indicate, again, the Baseline (B) and coloured bars and data points indicate the Test phase (T) for each treatment: pink and circles for “Social Control”, and blue and squares for “Social Treated. In **a** and **c**, females are represented by solid lines and males by dashed lines. Asterisks indicate statistical significance: **p < 0.01 and ****p < 0.0001 (colour figure online)

Male and female test fish spent equal time freezing upon sight of a non-alarmed shoal (planned comparisons: Male Social Control vs Female Social Control z = 0.886, p = 0.6443), but male test fish spent significantly more time freezing than female test fish upon sight of an alarmed shoal (planned comparisons: Male Social Treated vs Female Social Treated: z = − 2.869, p = 0.0099). Moreover, both male and female test fish showed a significant increase in freezing response upon sight of the alarmed shoal in comparison with the control group (planned comparisons: Male Social Treated vs Male Social Control z = 10.110, p < 0.0001; Female Social Treated vs Female Social Control z = 6.120, p < 0.0001). Moreover, both male and female freezing responses to alarmed conspecifics where significantly higher than during the Baseline phase (planned comparisons: Male Social Treated Baseline vs Male Social Treated Test: z = − 8.709, p < 0.0001; Female Social Treated Baseline vs Female Social Treated Test: z = -5.706, p < 0.0001). See Fig. 4a and b.

The erratic movement response of test fish was significantly affected by Treatment (F = 4.224, p = 0.0399) but not significantly affected by the Experimental phase (F = 1.090, p = 0.2964), the Sex of the test fish (F = 0.071, p = 0.7902), and the double and triple interactions of Experimental phase, Treatment and Sex (Experimental Phase*Treatment: F = 2.041, p = 0.1531, Experimental Phase*Sex: F = 0.000, p = 0.9907, Treatment*Sex: F = 1.956, p = 0.1619, Experimental Phase*Treatment*Sex: F = 0.003, p = 0.9547).

Male and female test fish did not differ in erratic movement response upon sight of an alarmed shoal (planned comparisons: Male Social Test vs Female Social Test: z = − 0.494, p = 0.8285), nor was there a difference between male and female control groups (planned comparisons: Male Social Control vs Female Social Control z = 0.784, p = 0.6716). Likewise, there was no significant difference of male and female test fish between the treatment with an alarmed shoal and the control treatment (planned comparisons: Male Social Test vs Male Social Control z = -0.339, p = 0.8626; Female Social Test vs Female Social Control z = 0.931, p = 0.6716). Moreover, both male and female erratic movement responses were similar across treatments between Baseline and the Test phase (all p-values > 0.05). See Fig. 4c and d.

Together, our observations demonstrate that when males and females were not directly exposed to the alarm substance but were exposed to the sight of an alarmed shoal, they both triggered freezing, but males at a significantly higher intensity than females. Contrastingly, males and females spent similar amounts of time in erratic movement and their erratic movement response was not triggered by the alarmed shoal, remaining very low.

## Discussion

Our study demonstrates that zebrafish use social information about predation threats to decide whether a threat is present or not and to, subsequently, modulate an intensity threshold of fear responses. Individual test fish, either males or females, adjust the intensity of their fear responses to the presence or absence of an alarm substance according to whether a shoal of four conspecifics has detected a similar threat or not. However, this adjustment is made only for freezing behaviour, not for erratic movement, and males and females respond at different thresholds.

Firstly, we show that both males and females respond to buffering and social contagion of fear. When they detect the alarm substance, they use the unalarmed responses of conspecifics to reduce the intensity of their own defensive response, decreasing the time spent freezing (social buffering). When they do not detect the alarm substance, they use conspecific alarmed responses to trigger their own fear response, increasing the time spent freezing (social contagion). These results agree with other studies investigating social buffering and social contagion of fear in zebrafish (Hall and Suboski 1995; Faustino et al. 2017; Fernandes Silva et al. 2019; Mathuru et al. 2017; Suboski et al. 1990), confirming that in threatening situations zebrafish use conspecific fear behaviours to modulate the intensity of their own fear responses.

Secondly, we show sex differences in social information use in threat detection. Males spent significantly less time freezing in social buffering and significantly more time freezing in social contagion than females. In social buffering, it was the test fish that received the alarm substance, and by reducing freezing in such situations, there is a greater probability of missing a real attack by a predator than of responding to a false alarm, and males, as higher risk-takers, risked substantially more than females, as we predicted. Males dropped their passive defence strategy to baseline levels, while females only reduced it, remaining above baseline levels. In social contagion, on the other hand, it was the shoal that received the alarm substance, making the probability of false alarms to the test fish greater than the probability of misses. Here, the risk of not responding to a real threat was reduced, but males still increased their defensive behaviours more than females, contrary to our prediction.

Male zebrafish have been reported to be more risk prone than females (Roy et al. 2017), and they also engage more in territorial and courtship behaviour (Spence et al. 2008). Hence, males may often need to move to a different site to acquire and defend a territory or to search for potential mates. Such relatively unfamiliar environments may impose selection pressures on males to rely more on information from resident conspecifics to optimize their predator evasion. Therefore, zebrafish males may be more sensitive to conspecific fear responses, both in buffering and contagion situations, because they are often more in need of that information.

Concerning females, in species in which they have high energetic demands imposed by pregnancy, lactation and parental care, they are more risk averse, thereby reducing the risk of predation and energetic shortfall, which could be fatal to themselves and their offspring (Reader and Laland 2000; Smolla et al. 2019). However, zebrafish females are not constrained by these behaviours, nor by the need to defend territories and search for mates (Spence et al. 2008), and therefore may need to rely less on conspecific fear responses to update their own assessment of risk. This implies that females rely more on chemical signals from conspecifics to assess risk, while males rely more on visual cues. This is, however, surprising because females express fewer chemosensory receptors in their olfactory epithelium than males (Wang et al. 2020), which should make them more reliant on visual information about conspecific fear behaviours than males. Alternatively, the alarm substance may be a truly safer source of information, as it is usually detected earlier than visual cues and can be detected at a safe distance, while visual information needs high proximity to conspecifics and predators (Stephenson 2016). If so, by relying more on chemical signals than visual cues from conspecifics, females are in fact following the safer strategy.

Thirdly, we show that zebrafish do not use conspecific fear responses to modulate the intensity of their erratic movement behaviour, which is always performed in much lower intensity than freezing (reaching an average maximum intensity of 20%, while freezing reached 80%). In social buffering, freezing decreases and in social contagion, freezing increases. In both, erratic movement does not change, remaining low, and does not vary between sexes. These patterns suggest that zebrafish respond to threatening situations with highly stereotyped erratic movement behaviour and subsequently adjust their predator defence strategy through the time spent freezing. On the other hand, there is also the possibility that other factors such as the size of the experimental tank and the absence of an escape route, such as the presence of a shelter or gravel in the bottom of the tank, may have affected the dynamic of the erratic movement response. In future studies, the use of larger tanks as well as the use of more behavioural and/or physiological (e.g. cortisol) measures of stress could be used to allow a better understanding of these effects.

Previous studies of zebrafish defensive/fear responses have not reported sex differences, but this is not surprising given that these experiments were performed using either only males (Faustino et al. 2017) or groups of mixed-sex fish (Suboski et al. 1990; Hall and Suboski 1995; Mathuru et al. 2017). Sex differences in zebrafish behaviour have been reported only in other contexts (Genario et al. 2019), such as territoriality (Spence et al. 2008), shoaling decisions (Ruhl and McRobert 2005), lateralization (Ariyomo and Watt 2013), activity levels (Tran and Gerlai 2013), anxiety-like behaviour (Fontana et al. 2019) and courtship behaviour (Spence et al. 2008). Hence, our study shows, for the first time, sex differences in zebrafish in the use of social information about predation threats. We can exclude the possibility that these sex differences could have resulted from differences in how males and females were raised and treated during data collection. Developmental conditions are known to affect the perception of risk in guppies (Elvidge et al. 2014) and the social context (e.g. shoal size or sex composition, Piyapong et al. 2010) is also known to affect fish responses to predation threats. However, our test fish were of the same age and were raised and tested in the exact same conditions.

In conclusion, our study demonstrates that a sexually dimorphic defensive/fear response occurs in zebrafish under threatening conditions. These sex differences may have been selected because zebrafish males’ life-history strategies are of higher risk than females. Males, by taking more risks, may end up facing predators more often. However, the benefits are also higher because adjusting a fear response more than females allows males to save more energy when the predator is indeed absent or to respond more quickly when it is present. This could be advantageous because it allows males to allocate more time and energy for competitive activities with other males, such as competition for territories and females. Because these life-history strategies are not unique to the male zebrafish, our findings may provide a broader view of how sex determined life-history strategies can modify the use of social information about predation threats and other contexts.

## Supplementary Information

Below is the link to the electronic supplementary material.

Supplementary file1 (XLSX 289 kb)

## Data Availability

The data supporting this article is provided as supplementary material.
